# Metals and Alloys

**Published:** 1862-08

**Authors:** B. Wood


### METALS AND ALLOYS.

BY DR. B. WOOD.

(Continued from Vol. XVI., p. 216.)

CADMIUM.—In individualizing the metals, I take up this first, seeing that it is little noticed in the books. Its properties appear to have been less clearly determined by chemists and metallurgists than of most other metals. Discrepancies exist among authors in respect to the metal, while some of its most remarkable, if not most useful, properties are not noticed by them, even when they are explicit and elaborate as to similar properties possessed by other metals. Our works on chemistry treat of cadmium very briefly, as of little importance, and one of the latest says it "has no practical value in the arts"—but further investigation might have discovered it to possess qualities rendering it useful in the arts, as well as interesting to science.

The melting point of cadmium is variously stated by authors. Some place it, indefinitely, "below a red heat." Overman, in his Treatise on Metallurgy, marks it at 550° Fahr., and indicates 600° as the temperature at which the metal volatilizes. Brande, Dict. Science and Art, says, "it fuses and volatilizes at a temperature a little below that at which tin melts." Webster, Manual of Chemistry, states that "it melts and volatilizes by a heat not much greator than that required to vaporize mercury." (Being according to the one author below 440°, and according to the other above 600°.) Most of our chemical text-books put its melting point at 442° F. (from Stromeyer.)

The heat at which it melts is too high for measurement by the mercurial thermometer. Melted under similar conditions by the side of other metals, it appears to require for its fusion nearly the same heat as lead. It is somewhat later in melting, but then it appears to congeal a little the later (which may be due to a difference in the conducting power of the two metals.) I should, therefore, place its melting point, in round numbers, at 600° Fahr., that of lead being placed by different authors at 590°, 600°, and 612°.

Cadmium, in its general characters, has a greater resemblance to tin than to other metals. It has less luster, tarnishes more readily in the atmosphere, is considerably harder, and requires a considerably higher heat for its fusion. It has a sort of milk-white, glistening hue, approaching a silver-white, with a bluish tinge, something like zinc. Melting about the same as lead, a little above that heat, or at a dull red heat it volatilizes, giving off orange-colored fumes; at a higher heat it flashes and detonates, and if the heat be still raised, it bursts into flame with an explosion. It is perfectly malleable and has considerable tenacity. In flexibility it is inferior to tin, ranking with lead. It dissolves rapidly in nitric acid, is acted on fully by muriatic acid, and very slightly by sulphuric acid. It tarnishes at once in strong solution of caustic potash, but the *solvent* action of this menstruum upon it appears to be very feeble.

Electrically, it is highly positive with respect to gold and silver. With some metals it appears to have little affinity; with others its affinity is very strong.

Its volatility renders its combination with the less fusible metals somewhat difficult under ordinary circumstances, though, perhaps, not more so in most cases than is the case with zinc.

Hardness, 5 ; Flexibility, 5 ; Malleability, 5 ; Fusibility 6. (See Scale, pp. 215, 216, vol. xvi.)

Of the general properties of cadmium as an ingredient in alloys, Overman says:

"Cadmium is very soft and malleable, and still all its alloys are brittle. Its combinations are not distinguished for fluidity." Again, "The combinations of platinum, copper, and other metals with cadmium are brittle and hard." The cause of this he ascribes to "its volatile nature and want of affinity," which, recurring to the subject, he accounts for thus:

"When it is melted with any other metal, there is a tendency on its part to evaporate; the slight affinity of cadmium for other metals causes a separation of its atoms from those of the other metal, and no intimate union can be formed. If, therefore, the alloy cools, there are spaces between the crystals which have been occupied by the expended atoms of cadmium, and in cooling these are filled again; this causes brittliness."—*Treatise on Metallurgy, p.* 465.

This is mainly correct in respect to *some* of the combinations of cadmium, such as the particular instances cited, but by no means of "all."

Other authors, although less explicit, ascribe a similar general character to the metal, but without so much as noticing the remarkable exceptions to the assumed rule. In *Berthier's Traité des Essais*, it is stated that "most of the alloys of cadmium are brittle," the individual instances there cited being particularly characterized as "brittle," and no mention made of others. The combination with mercury is thus described: "Cadmium unites with great facility with mercury, even when cold. The amalgam is a silver white, and texture granular and crystalline. It can be obtained in octohedrons. It is hard and very fragile. Its density is greater than that of mercury. It freezes at 75 [centigrade.] It contains 0.217 of cadmium."

Combined in these proportions, the compound will indeed be comparatively fragile; but one might be led to infer from this description that the metals combine in no other proportions. I have seen this particular formula cited by other authors when speaking of the combination of cadmium and mercury, but without any allusion to other compounds of these metals; although they unite with facility in other proportions forming amalgams, or rather *alloys* remarkable for tenacity, and particularly noteworthy as contrasted with the compounds of mercury with other metals.

While it has been assumed, as a general rule, as above quoted, that the combinations of cadmium are not distinguished for fluidity, I have not found its fluidifying properties in respect to certain metals and alloys noticed in any work. *Some* of its alloys indeed are not remarkable for fusibility, but rather for the reverse; such are its alloys with silver, antimony, and mercury, their melting point being but little lower, or even higher, than that indicated by the mean of their constituents. But others are much more fusible than the mean, as its alloys with lead, tin, copper, bismuth, etc. In certain instances it manifests this property in the most extraordinary degree, and it is singular that it should have been altogether overlooked. Bismuth holds a high rank among metals for its property of promoting fusibility in alloys, as is particularly remarked in all our text-books on chemistry and metallurgy. But in some combinations, cadmium displays this property still more decidedly, as will be seen when we come to particularize alloys.

As to the brittleness which cadmium is said to communicate when combined with "any other metal," the facts are, *some* of its alloys, even with malleable metals, *are* "brittle." But others are highly tenacious and malleable. Its alloys with gold, platinum, and copper afford instances of the former; its combinations with lead, tin, and to a certain extent with silver and mercury, are examples of the latter. An alloy of two parts silver and one part cadmium is malleable and very hard and strong; with equal parts it is also malleable, but has less tenacity; but in the proportions of two parts cadmium and one part silver, the alloy is brittle. Equal parts of cadmium and mercury form a tough and highly malleable compound; and in the proportions of even two parts of mercury to one of cadmium, the result is nearly equal in malleability with the other, but possesses less strength. This is remarkable, considering the extreme fragility of most amalgams. Thus a mixture of two or three parts tin with one part mercury almost crumbles to pieces in handling; equal parts being still more fragile.

Cadmium has been little used in connection with dentistry. Its expensiveness excludes it from the laboratory, where, but for this, it would be of value in the formation of dies. It is mentioned as a component in Dr. Blandy's "Cheoplastic" metal, to add greater rigidity to the alloy. It was recommended some years ago by Dr. Evans, of Paris, as a component, in place of silver, in connection with mercury, for an amalgam for filling teeth, but fell into disuse—an alloy of silver and tin being now generally used for this purpose.

In another number some of the alloys of this metal will be particularized.

*July* 16, 1862.

(To be continued.)

